# A novel variable delay Go/No-Go task to study attention, motivation and working memory in the head-fixed rodent

**DOI:** 10.12688/f1000research.2-125.v2

**Published:** 2014-03-19

**Authors:** Samuel D Dolzani, Shinya Nakamura, Donald C Cooper

**Affiliations:** 1 Institute for Behavioral Genetics, University of Colorado, Boulder, CO, 80309, USA; 2 Department of Psychology and Neuroscience, University of Colorado, Boulder, CO, 80303, USA

## Abstract

In order to parse the causal elements underlying complex behaviors and decision-making processes, appropriate behavioral methods must be developed and used in concurrence with molecular, pharmacological, and electrophysiological approaches. Presented is a protocol for a novel Go/No-Go behavioral paradigm to study the brain attention and motivation/reward circuitry in awake, head-restrained rodents. This experimental setup allows: (1) Pharmacological and viral manipulation of various brain regions via targeted guide cannula; (2) Optogenetic cell-type specific activation and silencing with simultaneous electrophysiological recording and; (3) Repeated electrophysiological single and multiple unit recordings during ongoing behavior. The task consists of three components. The subject first makes an observing response by initiating a trial by lever pressing in response to distinctive Go or No-Go tones.  Then, after a variable delay period, the subject is presented with a challenge period cued by white noise during which they must respond with a lever press for the Go condition or withhold from lever pressing for the duration of the cue in the No-Go condition. After correctly responding during the challenge period (Challenge) and a brief delay, a final reward tone of the same frequency as the initiation tone is presented and sucrose reward delivery is available and contingent upon lever pressing. Here, we provide a novel procedure and validating data set that allows researchers to study and manipulate components of behavior such as attention, motivation, impulsivity, and reward-related working memory during an ongoing operant behavioral task while limiting interference from non task-related behaviors.

## Introduction

The use of operant behavioral tasks that utilize the head-immobilized condition in rodents have some advantages over tasks that occur under freely moving conditions by limiting the range of possible non task-relevant behaviors and distractors. While the head-restrained behaving rodent has been examined for many years, the relevant literature is minimal in comparison to similar behavioral tasks involving freely moving animals. Head immobilization is a traditional and common method in electrophysiological studies utilizing behaving monkeys, in which skilled intentional forelimb movements (e.g. grasping) are widely used as conditional responses. Compared with monkey studies, despite a growing number of studies that utilize head-fixed rodents, very few experiments have assessed psychological and physiological processes simultaneously occurring during operant behavioral tasks that require intentional skilled movements (e.g. lever pressing) as conditional responses in the awake, head-restrained rodent ^1, 2^.

The Go/No-Go task, a canonical paradigm in animal behavior and psychology, requires the subject to initiate an operant conditioned response during one stimulus (CS+) and withhold from such a response during the presence of the opposite type of stimulus (CS-) ^1, 3^. Variations of the Go/No-go task have long been applied to electrophysiological, lesion and pharmacological studies ^4– 7^. This type of behavioral task has a high level of construct validity and clinical utility when studying the brain motivation/reward pathways and behaviors such as impulsivity. Impulsivity and motivational deficits are complex behavioral phenotypes implicated in a number of psychiatric disorders including attention deficit hyperactivity disorder (ADHD), mania and depression associated with bipolar disorder, schizophrenia, pathological gambling, borderline personality disorder, and substance abuse ^3^.

The Go/No-Go task we developed allows for simultaneous measurement of the subject’s motivational level, impulsivity and short-term memory. The task consists of four periods: initiation, delay, challenge and reward. First, rats are required to press the lever in response to distinct Go or No-Go tones to initiate a trial (initiation). Performance during this period can be used as a measurement of motivational level. After initiating a trial, a variable delay follows and the rats must maintain information about the cue (tone frequency). After the delay, rats must make a correct Go or No-Go response according to the preceding tones (challenge). A delay period can be used to assess short-term memory and performance during the challenge period can be used as a measure of impulsivity. Finally, a reward tone of the same frequency as the initiation tone is presented following a correct response during challenge period. Data obtained from these studies provide the opportunity for development of novel future pharmacotherapies and behavioral interventions ^8^.

The head-immobilized rodent Go/No-Go task we have developed is a procedure that provides the framework for performing repeated electrophysiological recordings and simultaneous molecular and pharmacologic interventions during ongoing operant behavioral tasks. In addition, this technique allows for accurate stereotaxic localization during electrophysiological recordings, daily anesthesia-free recordings in various brain regions, rapid electrode repositioning during chronic recording sessions, and high quality stabilized recordings ^8^. With the advent of optogenetics, the head-restrained procedure allows for these recordings to be coupled with cell-type specific silencing and activation, free from electrophysiological artifacts ^9^.

## Methods

### Animals

Four male Sprague Dawley rats and 28 male Long Evans rats (8 weeks and 200–300 g at time of surgery), bred at the University of Colorado Boulder, were used for all experiments. Animals were maintained on a 12 h reverse light/dark cycle (lights off at 07:00). The behavioral experiments were conducted during the dark period. Animals were singly housed and water-restricted, while food was available *ad libitum* for the duration of the experiment. All procedures were in compliance with animal care standards set forth by the Institute for Animal Care and Use Committee at the University of Colorado Boulder.

### Surgical preparation

Animals were stereotaxically affixed with stainless steel head-caps prior to any behavioral training. Rats were deeply anesthetized with an intraperitoneal injection of ketamine-xylazine (Sigma-Aldrich; 80 mg/ml ketamine/6 mg/ml xylazine) and restrained on a custom stereotaxic device (Old School Industries, CO, USA). Head fur was shaved and a rostro-caudal incision was made from the region roughly 10 mm anterior to bregma, extending 5–7 mm posterior to lamba. Connective tissue on the skull was carefully removed and temporal muscles were retracted from the dorsolateral region of the skull. Vasculature was cauterized using 30% hydrogen peroxide (Sigma-Aldrich, USA) and quickly rinsed with sterilized saline. Eight self-tapping screws (Small Parts, IN, USA) were implanted on the dorsal surface of the skull and four screws were implanted on the lateral region of the skull to confer head-cap stability and strength. After screws were implanted, the skull was sterilized using povidone-iodine (9%) (Sigma-Aldrich, USA) then desiccated with sterile cotton swabs. A thin layer of radiopaque glass ionomer restorative cement (GC America Inc., USA) was applied to the skull and around the base of the reinforcing screws to increase adhesion strength between the skull and head holder cap. After allowing the radiopaque cement to set, two horizontal stainless steel head restraint bars were stereotaxically positioned directly above the exposed dorsal region of the skull (5 mm anterior to bregma and 3 mm posterior to lamda). Bars were positioned approximately 5 mm above the dorsal surface of the skull and acrylic dental cement (Stoelting, USA) was applied between the head restraint bars and the skull. After implantation of screws and head restraint bars, surrounding areas of the skull were disinfected using povidone-iodine and antibiotic ointment (Sigma-Aldrich, USA) was applied around the site of incision. Animals were given 7 days to recover with food and water available *ad libitum*.

### Experimental setup

All behavioral training was conducted using a custom head-restraint system (Old School Industries, CO, USA) in a red acrylic box. A reward-delivery nozzle, lever, sensor systems for detecting licking movements and lever-presses, and a speaker were placed around the head-restraint stage. All computer software used for the experiments was customized using LabVIEW (National Instruments, USA) and a digital input/output interface (USB-6008, National Instruments, USA) was used for controlling all devices.

## Behavioral conditioning procedure

### Habituation to training device

After animals recovered from surgery, they were handled for 3–5 days and allowed to explore (20 min/day) the operant conditioning boxes that they were restrained within for the remaining duration of the experiment. The purpose of this was to familiarize the animal with the experimental environment and reduce context-related stress and excitability. Subjects were head restrained on an ergonomically designed custom head-restraint system (Old School Industries, CO, USA) for 60 minutes/day during the habituation period of the training procedure. During this time period, subjects were administered 0.3 M sucrose (Sigma-Aldrich, USA) non-contingently through a reward delivery nozzle positioned near the mouth. Approximately 40 µl sucrose was delivered every 10 seconds (10 s inter-trial interval (ITI)) over the course of the 60 minute habituation session, yielding a total reward delivery of 15 ml/60 minute session.

### Acquisition of lever-pressing and lever/tone association

**Figure 1.**
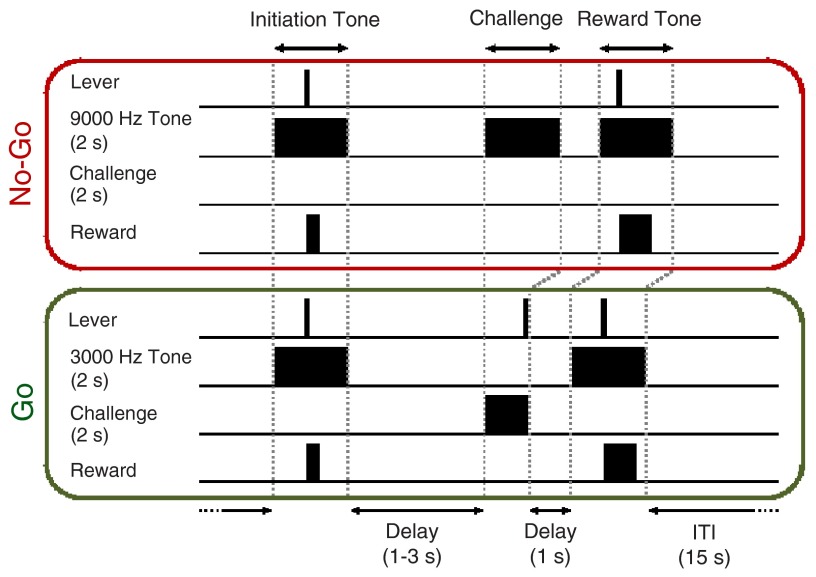
Schematic showing the head-fixed Go/No Go task under a No-Go trial and Go trial. Lever presses, initiation tone, challenge period, reward tone, and sucrose reward delivery components are shown for both trials. The black boxes represent the relative timing of the rat’s response (lever press) in response to the auditory cues. ITI: inter-trial interval.

Following successful sucrose consumption under non-contingent reward delivery conditions, subjects were trained to press a right forepaw lever to receive sucrose reinforcement ( Figure 1). This stage of operant behavioral training was intended to facilitate the contingent relationship between lever pressing and reward delivery. This phase of training persisted until the subject responded with >1000 lever presses for 2–3 consecutive days. The next phase of training was the Lever/Tone association phase during which subjects learned to lever press in the presence of an audible tone. The two tones randomly presented were either 3000 Hz or 9000 Hz, which corresponded to the same frequencies used in the Go and No-Go constituents of the later stages of the training procedure. This stage of training began with a 5 s tone presentation with 8 µl sucrose delivered for every lever press occurring during the tone (15 s ITI). Subjects were switched to the next stage of training after >70% correct responding during the presence of the tone. The next phase of training was a reduction in the duration of the tone presentation (5 s to 3 s). After achieving >70% correct responding using these parameters, tone duration was finally decreased to 2 s with a single lever press contingent sucrose delivery of 40 µl. The final phase persisted until subjects achieved >80% correct responding for 2 consecutive sessions.

### No-Go acquisition

First, subjects were trained to perform the No-Go constituent of the behavioral task after successfully completing the Lever/Tone association phase ( Figure 1). During No-Go trials, the subject was required to initiate a trial by lever pressing in response to 3000-Hz, 2-s tone (initiation tone). Correct trial initiation yielded an 8 µl sucrose delivery. Following the initiation tone response, a variable delay (1–3 s) was followed by a 2-s white-noise presentation (challenge period). This was the specific epoch in which the subject must withhold from lever pressing (No-Go). Following successful suppression of lever pressing during the challenge period, the 2-s 3000-Hz tone was presented again (reward tone). A second lever press was required during the presence of the final reward tone and a larger 40 µl sucrose was delivered in response to this lever press. Following a 15-s ITI, the next initiation tone was presented. If the subject failed to initiate a trial or withhold from lever pressing during the challenge period, the trial was terminated and a 15-s ITI followed. No-Go acquisition training proceeded until the subject responded at >80% correct challenge period responding for two consecutive days before moving to the Go condition training.

### Go acquisition

After successful acquisition of the No-Go constituent of the training procedure, subjects began daily 90 minute Go training sessions ( Figure 1). In a Go trial, subjects must lever press to initiate a trial during the presence of a 9000-Hz, 2-s initiation tone. 8 µl of sucrose was delivered in response to the lever press. Following initiation of a trial, a 2-s white noise (challenge period) was presented after a variable delay (1–3 s). The subject must lever press (Go) during the challenge period. Lever pressing during the challenge period terminated the white noise and was followed by a 2-s final reward tone (9000 Hz). The larger 40 µl sucrose reward was only delivered if the subject correctly lever pressed during the reward tone. In the early stage of Go training, a white noise signal of indefinite duration was used for the challenge period. As subjects learned the association between the 9000-Hz initiation tone and lever pressing during the challenge period their reaction time decreased and the challenge period duration was decreased to 2 s. Subjects were trained using these parameters until they achieved >80% correct responding during the 2-s challenge period for 2 consecutive days before a randomized within session Go/No Go condition began (data not included).

## Results

**Figure 2.**
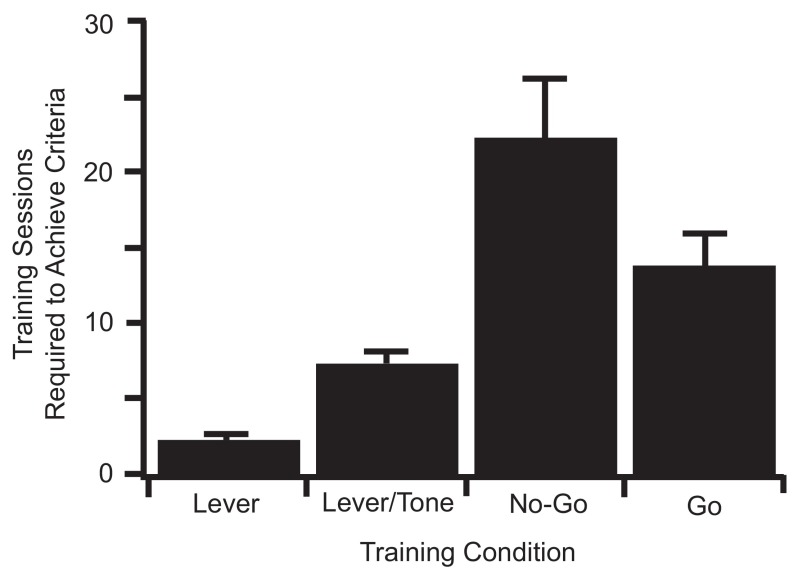
The average and standard error of the mean training sessions required to achieve specified criteria for acquisition of each head-fixed training condition of the behavioral task is shown. Lever: n=24, Lever/Tone: n=20, No-Go: n=14, Go: n=13.

**Figure 3.**
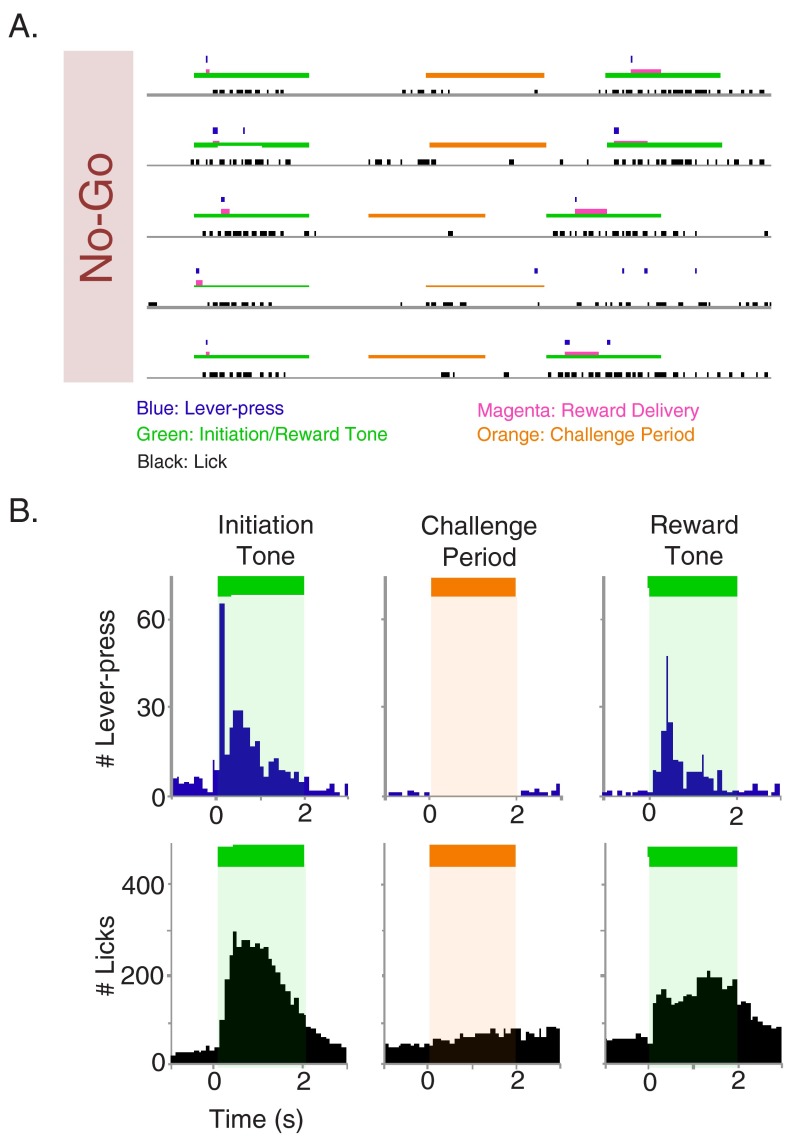
Raster plot of five consecutive No-Go trials ( **A**) and histograms of lever pressing and licking during initiation tone, challenge period, and reward tone ( **B**). Green bar: initiation tone and reward tone, orange bar: challenge period, magenta bar: reward delivery, black: lick, blue: lever press. (Histogram bin size=100 ms).

**Figure 4.**
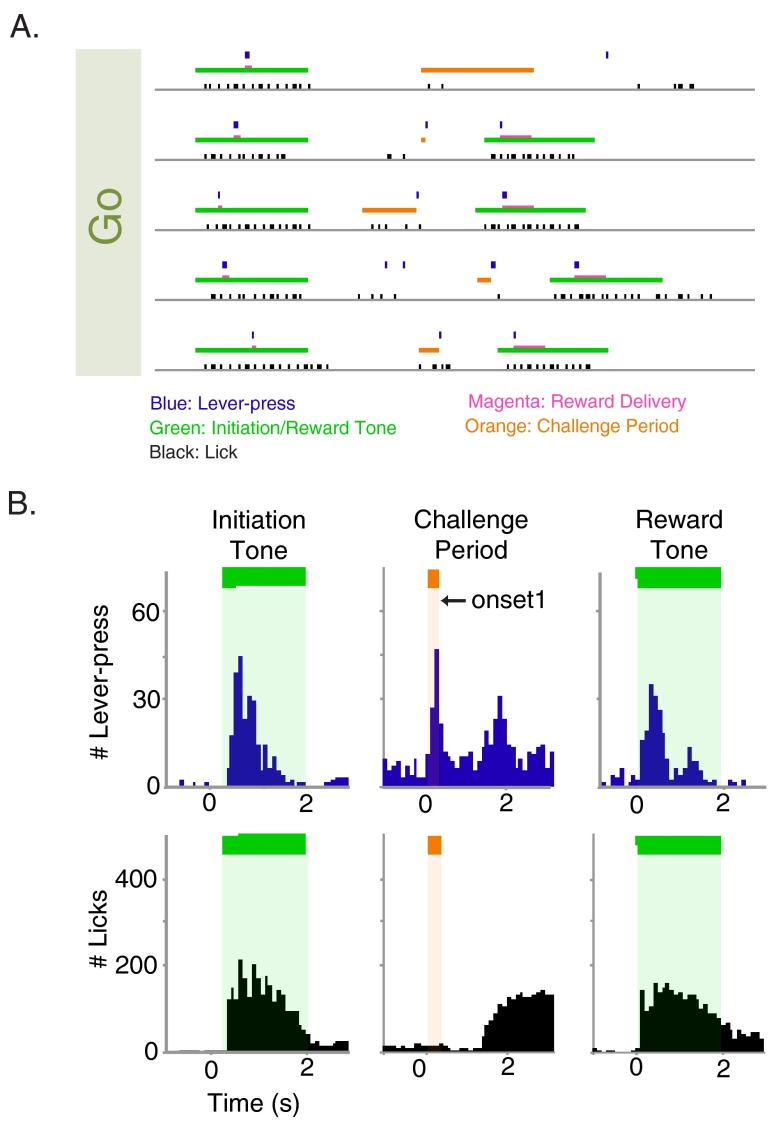
Raster plot of five consecutive Go trials ( **A**) and histograms of lever pressing and licking during the initiation tone, challenge period, and reward tone ( **B**). Green bar: initiation tone and reward tone, orange bar: challenge period, magenta bar: reward delivery, black: lick, blue: lever press. (Histogram bin size=100 ms). The second peak on the histogram of lever pressing during the challenge period represents lever presses during the final reward tone. Note the emergence of non cued lever presses and licking during the challenge period that represents the idiosyncratic variable initiation during the Go challenge tone and subsequent reward tone delivery under the Go condition.

**Figure 5.**
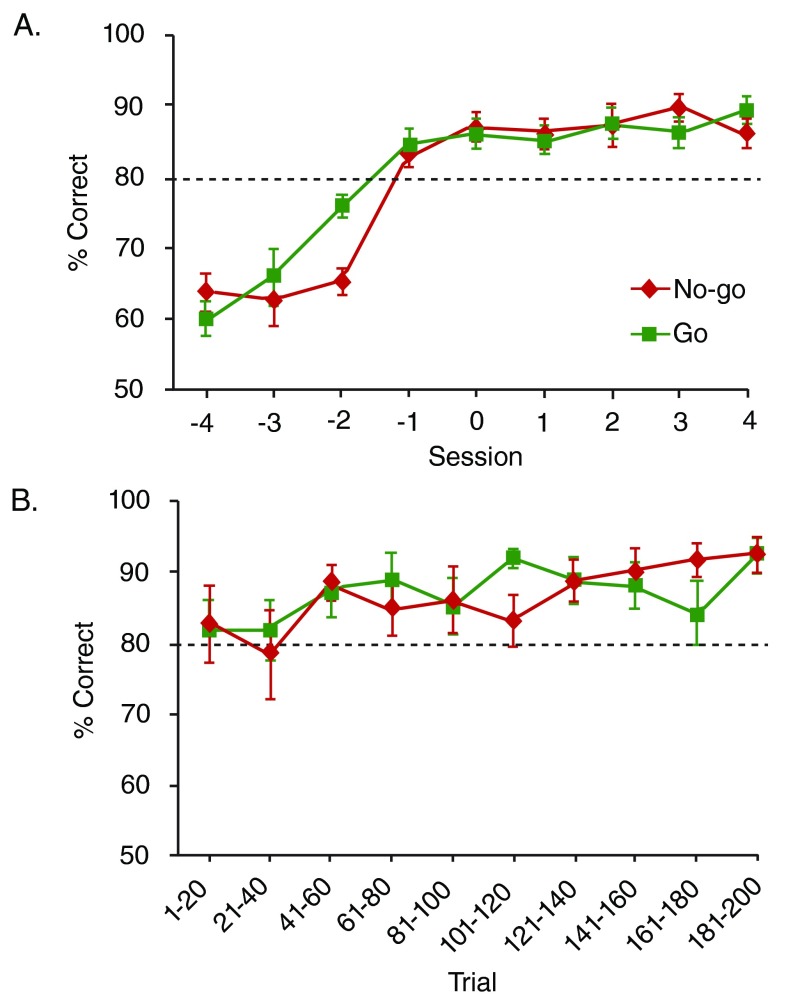
Stability of the task performance between ( **A**) and within ( **B**) sessions after reaching criteria. **A**. Session 0 represents the day when the rats (n=10) achieved the criteria (two consecutive training sessions with >80% (dashed horizontal grey line) correct challenge period responding). **B**. Within session challenge period responding for 200 trials, divided into 20 trial bins, is shown for the fourth training session after achieving criteria. Error bars indicate the standard error of the mean.

Subjects rapidly acquired the ability to consume sucrose delivered non-contingently through a reward delivery nozzle. Following acquisition of sucrose consummatory behavior, subjects quickly learned (<3 days) an operant response by lever pressing (Lever) to receive sucrose reinforcement ( Figure 2). On average, subjects reached the specified criteria of >1000 presses/90 minute training session in <3 consecutive daily training sessions (n=24). Next, subjects reliably acquired operant lever pressing behavior during the presence of a tone (Lever/Tone association) to receive sucrose reinforcement ( Figure 2). The Lever/Tone association stage of training required an average of 8 training sessions to achieve the criteria of >70% correct responding for two consecutive days (n=20). This requisite stage of training was intended to facilitate the association between tone presentation, lever pressing, and concomitant primary reinforcement. No-Go training (No-Go) followed the Lever/Tone association training ( Figure 2). The No-Go constituent of the behavioral task required an average of 22 daily training sessions to achieve criteria of >80% correct challenge period responding (n=14). Finally, subjects were trained to perform the Go constituent (Go) of the Go/No-Go task ( Figure 2). This component of the task required an average of 14 daily training sessions to achieve a criteria of >80% correct 2 s challenge period responding for 2 consecutive days (n=13). Raster plots of five consecutive representative No-Go trials ( Figure 3A) and histograms of lever pressing and licking during the initiation tone, challenge period, and reward tone are shown ( Figure 3B). The histograms represent cumulative responding across a 90 minute training session. Subjects lever pressed in response to an initiation tone and received an initial small sucrose reinforcement (8 µl), withheld from lever pressing (No-Go) during the challenge period, and emitted a final lever press during the final reward tone to receive a larger 40 µl sucrose reinforcement. One example of a failed No-Go trial is shown (4 ^th^ trial on the raster plot). The subject emitted a lever press during the challenge period of the No-Go trial, resulting in a failed trial and no final 40 µl sucrose delivery. Raster plots of five consecutive Go trials ( Figure 4A) and histograms of lever pressing and licking during the initiation tone, challenge period, and reward tone are shown ( Figure 4B). Subjects initiated a trial by lever pressing during the initiation tone, emitted a lever press during the challenge period (Go), and lever pressed during the final reward tone to receive sucrose reinforcement. One example of a failed Go trial is shown (1 ^st^ trial on the raster plot). The subject failed to lever press during the challenge period of the Go trial and the final 40 µl sucrose reward was not delivered. Once the specified criteria of >80% correct responding for 2 consecutive days was achieved for No-Go and Go training sessions, subjects (n=10) maintained stable challenge period responding between subsequent training sessions ( Figure 5A). Similarly, subjects stably responded during the challenge period within individual No-Go and Go training sessions ( Figure 5B). The data set from all rats is presented.

Variable delay Go/No-Go task data Included are data files for the various behavioral task conditions. Each subfolder (Go, Lever Press Acquisition, Lever Tone Acquisition, and No-go) contains individual folders for the rats that underwent the specified task condition with a brief task sequence/results file and timestamp file named by the date of the training session. For each training session there is one task sequence/results file and one timestamp file. The files will retain their format when opened using Microsoft Excel (Windows, USA). Task sequence/results file: The far left column of this file states the trial number. The second column (Task Condition) states the behavioral task condition. The third column states the frequency of the presented tone. The fourth column lists either a 1 for a correctly performed trial or a 0 for an incorrectly performed trial. The fifth column lists the number of licks for the given trial. The sixth column lists the number of lever presses with the left lever, which was inactive for this experiment. The seventh column lists the number of lever presses with the right forepaw lever for the respective trial. The eighth column states if a correction trial was used (0 corresponding to no correction trial, 1 corresponding to correction trial). Timestamp file: The first pair of columns on the left represent the time that licking began and the time that it ended. The next two columns represent left lever on and off times. The third pair of columns represents the onset of right lever pressing and the offset of right lever pressing. The fourth pair of columns shows the onset and offset of white noise. The fifth pair of columns shows the onset and offset of the delivery of the final reward. The sixth pair of columns shows the onset and offset of the initiation tone. The seventh pair of columns shows the onset and offset of the final reward tone. The eighth pair of columns shows the onset and offset of the first reward that is delivered in the presence of initiation of a trial.

## Data availability

figshare: Variable delay Go/No-Go task data, http://dx.doi.org/10.6084/m9.figshare.654019 ^10^
