# Supplementary material for: A novel variable delay Go/No-Go task to study attention, motivation and working memory in the head-fixed rodent
Source: F1000Res. 2014 Mar 19;2:125. Originally published 2013 May 10. [Version 2] doi: 10.12688/f1000research.2-125.v2 (PMC3976106; doi:10.12688/f1000research.2-125.v2)
Supplement: Peer review history

# Peer review: Referee response for version 2

**Published:** 2014-03-21

**Authors:** Jeff Dalley

**Affiliations:** 1 ICCAM Cambridge, Department of Psychiatry, University of Cambridge, Cambridge, UK

I have read this submission. I believe that I have an appropriate level of expertise to confirm that it is of an acceptable scientific standard.

# Peer review: Referee response for version 1

**Published:** 2013-07-09

**Authors:** Jeff Dalley

**Affiliations:** 1 ICCAM Cambridge, Department of Psychiatry, University of Cambridge, Cambridge, UK

This is an interesting and well-conducted study on the development and validation of a novel, symmetrically-reinforced Go/No-Go task in rats. The novelty of this procedure presumably rests with head fixing the animals to limit the impact of extraneous behaviour, and the configuration of the task itself, which enables several component cognitive and behavioural processes to be independently assessed. One could imagine using such a task in a variety of settings to assess attention (e.g. based on Posner’s task), inhibitory response control, and working memory. However, this is not a radically new approach; for example, head restraint is often used to avoid the use of a general anaesthetic agent in MR and PET imaging and head restraint has been used for decades to facilitate neurophysiological experiments in non-human primates. Furthermore, restraint itself is a potent activator of the stress response which may have unintended consequences for brain-behaviour relationships. It would have been interesting to compare blood corticosterone in head-fixed and non-head-fixed animals. The reported behavioural findings suggest that sucrose was sufficient as a reinforcer but crucially, how stable was performance both within and between sessions? 

Overall, the article is clear and concise with methods that are fully transparent. Although the Title and Abstract reflect the content of the article it would be helpful to explain the novel aspects of the present protocol.

I have read this submission. I believe that I have an appropriate level of expertise to confirm that it is of an acceptable scientific standard, however I have significant reservations, as outlined above.

# Peer review: Referee response for version 1

**Published:** 2013-07-03

**Authors:** Kenichiro Tsutsui

**Affiliations:** 1 Department of Biology, Tohoku University, Sendai, Japan

This interesting paper describes the details of a go/no-go task designed for head-restrained rats. A detailed description about the training procedure is obviously beneficial for the biological community since the use of behaving rats is emerging in optogenetics and electrophysiology studies. I would like to ask the authors to consider the following points for the improvement of the paper. (All are “minor” comments.)

**1.** Go/no-go tasks have long been commonly used in behavioral psychology, and its application to behavioral electrophysiology with head-restrained monkeys is as early as in 1980s. The authors could briefly mention the history of go/no-go task in the 'Introduction' section. 

**2.** I am not entirely sure which part of the described go/no-go task is “novel”. Please point it out clearly in the manuscript which part of this protocol is different from the conventional go/no-go task procedure. 

**3.** How stable is the performance of a typical subject that has reached the criteria of training? It would be helpful to show the weekly or monthly performance of a representative subject after the completion of training.

I have read this submission. I believe that I have an appropriate level of expertise to confirm that it is of an acceptable scientific standard.
